# First-principles study of point defects at a semicoherent interface

**DOI:** 10.1038/srep07567

**Published:** 2014-12-19

**Authors:** E. Metsanurk, A. Tamm, A. Caro, A. Aabloo, M. Klintenberg

**Affiliations:** 1 Department of Physics and Astronomy, Uppsala University, Box 516, SE-75120 Uppsala, Sweden; 2 Intelligent Materials and Systems Laboratory, Institute of Technology, University of Tartu, Tartu, Estonia; 3 Los Alamos National Laboratory, Los Alamos, New Mexico 87545, USA

## Abstract

Most of the atomistic modeling of semicoherent metal-metal interfaces has so far been based on the use of semiempirical interatomic potentials. We show that key conclusions drawn from previous studies are in contradiction with more precise ab-initio calculations. In particular we find that single point defects do not delocalize, but remain compact near the interfacial plane in Cu-Nb multilayers. We give a simple qualitative explanation for this difference on the basis of the well known limited transferability of empirical potentials.

Nanostructured metallic multilayer composites (NMMC) are known to have superior mechanical properties as compared to standard coarse grained metals^1^ along with the ability to efficiently self-heal radiation damage^2^,^3^. The latter is crucial for the material to inhibit creep and swelling in harsh environments. Development and optimization of these materials requires profound understanding of the underlying mechanisms responsible for these properties.

Today state-of-the-art has reached to a point where it is possible to engineer bulk nanostructured bi-metal multilayers while having control over structural features at an atomic level leading to the ability of altering the mechanical properties and thermal stability of these materials in a most remarkable way^4^. Side-by-side to experimental work advanced modeling offers a complementary approach to gain insight into how these materials behave in extreme mechanical or radiation environments. Because the time and length scales these processes cover are exceptionally widely spread, starting from attoseconds and picometers for electronic effects and going up to meters and years in continuum mechanics, there is no single equation or model that currently can cover the full spectrum. Therefore only a multi-scale modeling approach allows for the prediction of properties and eventually the design of optimal NMMCs for future industrial and energy technology applications.

This study is concerned with the atomistic level of the multi-scale methodology. There has been considerable effort to model the structure and behavior of NMMCs in order to better understand the traits leading to the high tolerance to radiation damage. While the effects caused by irradiation are essentially macroscopic, these are still governed by changes at the atomic level that can often be traced back to properties of single point defects. Significant effort has been directed to identify possible lowest energy structures for the undamaged interfaces as well to describe the point defect properties such as configurations, formation energies, migration barriers and diffusion mechanisms near the interfacial plane. Previous studies have shown that some interfaces will host pairs of extended jogs instead of conventional point defects, i.e. vacancies and interstitial atoms. These delocalized defects have been shown to exhibit low formation energies and long-range interaction forces^5^. This results in more complex migration pathways and recombination mechanisms when compared to the bulk material^6^,^7^,^8^.

The studies described above were performed using atomistic modeling based on classical molecular dynamics and empirical interatomic potentials which play a crucial role in determining the outcome of the simulations. Fitting empirical models to ab-initio or experimental data always presents the challenge of obtaining good transferability to regions of phase space not used in the fitting process. For metallic systems the most widely used model is the embedded-atom method (EAM) developed by Daw and Baskes^9^,^10^. Although there are more complex alternatives, EAM is still relevant because of its simplicity and computational scalability while shown to be relatively accurate, especially for FCC metals.

There are two EAM potentials available for the copper-niobium system. The first, by Demkowicz et al^11^ (hereafter EAM1) uses a modified Morse function for the Cu-Nb interaction, and was fitted to dilute enthalpies of mixing, and bulk modulus and lattice constant of a hypothetical CuNb alloy in the B2 structure. The second, by Zhang et al^12^ (EAM2) uses a more flexible polynomial-like function and is fitted to enthalpies of mixing of special quasi-random structures over the whole composition range with the aim of correctly reproducing experimental thermodynamic data for this system.

In this work we first show that EAM1 and EAM2 give markedly different results for both the structure and energetics of point defects near the interface, and secondly we resolve this discrepancy using density-functional theory (DFT) calculations that do not rely on empirical parameters hence producing more reliable results. We finally discuss why some interatomic potentials may lead to erroneous characterization of the interfaces and how to possibly prevent this in future works.

## Results

**Figure 1:**
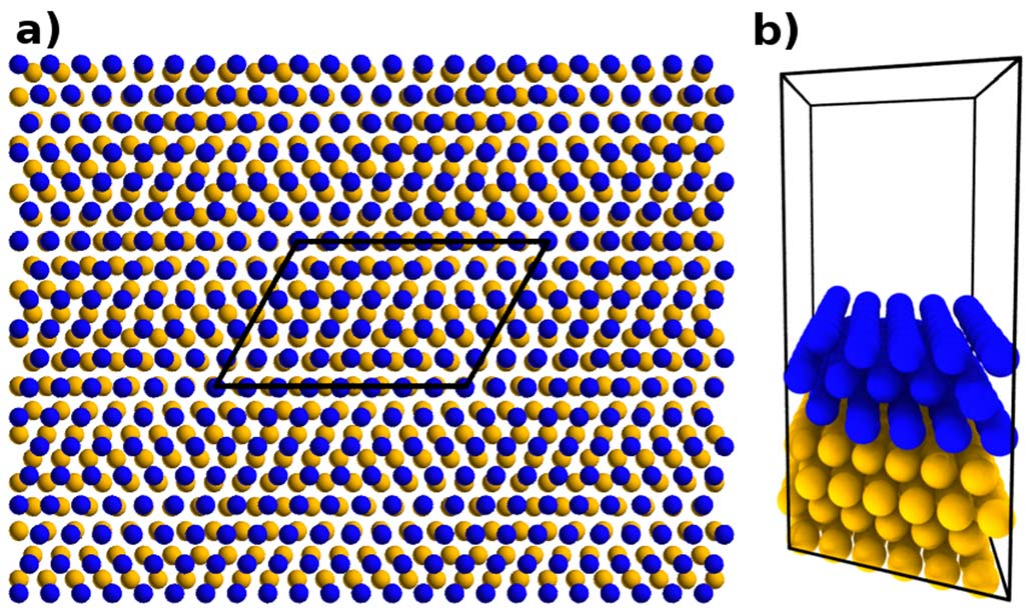
Constructing small quasi-unit cell for Cu/Nb interface in Kurdjumov-Sachs orientation. Blue spheres represent niobium and yellow spheres copper atoms. (a) The unit cell is “cut out” from a larger structure and has approximately same periodicity as the misfit dislocation intersections, that is the areas where copper and niobium atom positions coincide in x- and y-directions. (b) A 3D view of the simulation cell, where each layer consists of either 54 Cu or 40 Nb atoms. Total number of atoms is 336.

The structure of an interface can be described by specifying the orientation of the surface normal to it and two directions, one for each surface, that will be parallel when the interface is formed. It has been shown experimentally that copper-niobium interface forms predominantly in Kurdjumov-Sachs orientation^13^. Calculating the energies of such structures using DFT is a complex task because of the number of atoms needed to capture the characteristic features and periodicity of the interface, and hence the required computational effort, is large. The periodicity of the interface is defined by the misfit dislocation network and their intersections (MDIs)^14^. In the case of copper and niobium in KS orientation the distances between the MDIs are relatively small enabling this specific interface to be modeled using a quasi-unit cell appropriately sized for DFT. This cell is an approximation and not a true unit cell for the larger system. At this interface Cu {1 1 1} planes are parallel to Nb {1 1 0} and Cu 〈1 1 0〉 directions parallel to Nb 〈1 1 1〉. Unit cell size in aforementioned directions are 0.2556 nm for Cu and 0.2869 nm for Nb. The distance between MDIs in that direction is 2.3 nm corresponding to ≈ 9.0 replications of Cu and ≈ 8.0 of Nb unit cells while the strain for Nb layer is less than 1%. In perpendicular direction (Cu 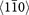) the unit cell sizes are 0.4427 nm and 0.8113 nm for Cu and Nb respectively and the distance between MDIs 1.33 nm. This corresponds to ≈ 3.0 replications for Cu unit cell and ≈ 1.6 for Nb. A row of Nb atoms have to be excluded to restore the periodicity. This resembles strain increments of 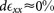, 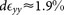 and 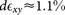 as compared to the original configuration. Whereas the quantitative values from present ab-initio calculations may not be comparable to those where the imposed strain is smaller, it does not affect the conclusions drawn about the structure of defects since the comparison between MD and DFT is performed using the same simulation cell. The setup is illustrated in Fig. 1.

In order to keep the calculations computationally feasible the number of atomic planes in the simulation cell perpendicular to the interface must be small. There are two choices to handle the periodic boundary condition in this direction, either to keep it in its present condition or add a vacuum layer. The former case corresponds to having infinite number of thin alternating copper and niobium layers and the latter to a single interface and two free surfaces. We opted for having a 1.65 nm layer of vacuum between the free surfaces. Unit cell vectors (in nm) for the resulting unit cell are *a_x_* = (2.3, 0, 0), *a_y_* = (0.75, 1.33, 0) and *a_z_* = (0, 0, 3.0) and it contains 216 Cu and 120 Nb atoms. Doubling the number of layers in the initial structure did not have significant effect on the relaxed structure and vacancy formation energy which decreased 20 meV thus confirming adequate convergence. While constructing a small unit cell using the method described above gives an appropriate representation of the undamaged interface, the challenge of finding ground state configurations of point defects remains. The ratio of lattice constants of Cu and Nb calculated using DFT and MD differ by less than 0.1% which allowed for calculating candidate structures using both EAM1 and EAM2 with molecular dynamics and subsequently relaxing these using DFT.

**Figure 2:**
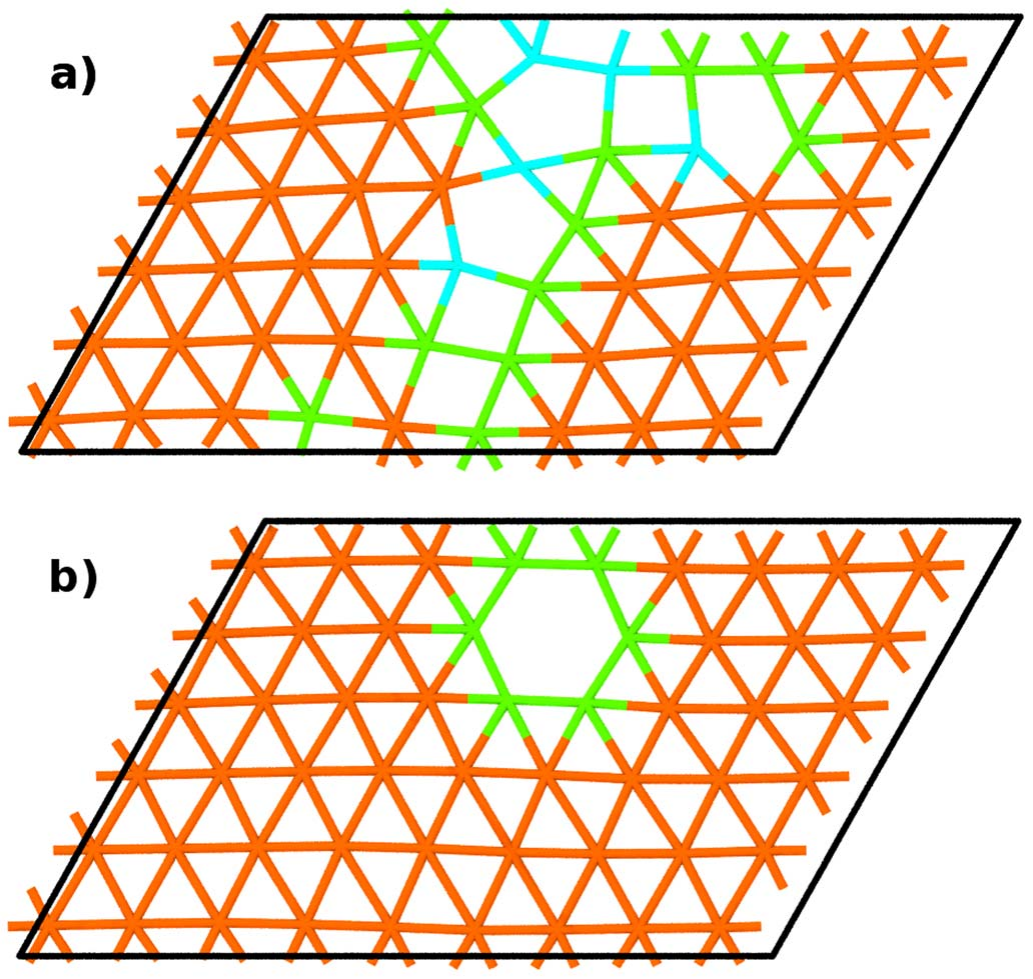
Top view of interfacial copper layer with single vacancy at the interface before and after relaxing using DFT. (a) The structure was obtained by relaxing the interface with EAM1 which results in delocalization of the defect and formation of 4- and 5-atom rings. (b) Relaxing in DFT yields single compact vacancy. Lines and colors represent the in-plane bonds and coordination numbers respectively with orange being 6, green 5, cyan 4. The position of vacancy coincides with the misfit dislocation intersection.

In order to i) check whether the vacuum layer or distortion of the true periodicity has any effect on the structure of the interface, ii) get input structures for DFT calculations and iii) assess whether relaxing the box has an important effect on the outcome of the results, we first performed different molecular dynamics simulations using both EAM1 and EAM2. First the initial system was quenched from 600 K to 0 K followed by energy minimization using conjugate gradient method. Next the copper atom with highest potential energy was removed and the process was repeated. A typical final structure as predicted by EAM1 is shown in Fig. 2a and is characterized by 4- and 5-atom rings as found in previous works^5^,^6^,^7^,^8^. Using EAM2 results in the formation of a compact single vacancy. Relaxing both the simulation box and atoms using EAM2 has minuscule effect on the formation energies while using EAM1 results in somewhat smaller energies. In either case the aforementioned structural features are not affected when compared to the case where the size of the cell is kept constant.

**Figure 3:**
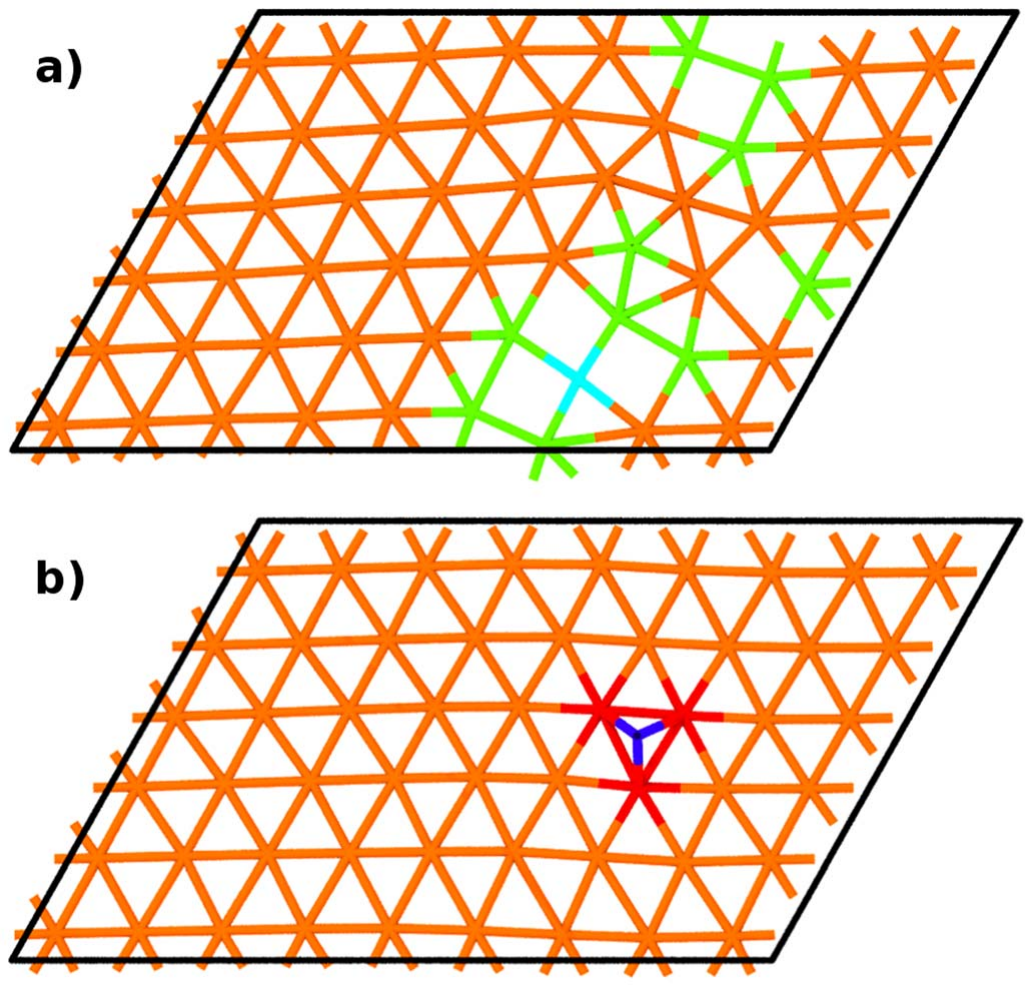
Top view of interfacial copper layer with added interstitial atom before and after relaxing using DFT. (a) The structure was obtained by relaxing the interface with EAM1 potential which results in delocalization of the defect and formation of 4-atom rings. (b) After relaxing in DFT a single interstitial between the interfacial copper and niobium layers at the location of an MDI will emerge. Lines and colors represent in-plane bonds and coordination numbers respectively, with red being 7, orange 6, green 5 and cyan 4 and blue 3.

The same procedure was carried out with a single interstitial copper atom which was inserted into the interface next to the MDI after initial energy minimization. Again using EAM1 results in delocalization of the defect (on Fig. 3a) while EAM2 produces a clear interstitial that resides between the copper and niobium layers.

Next all four structures were relaxed using DFT. The energy minimization was done using conjugate gradient method until the maximum force on an atom was less than 0.1 eV/Å while the energy difference between two sequential minimization steps was below 0.1 meV. The box size was kept constant for consistency and lower computational cost based on the fact that no change in structure and only negligible effect on formation energies was observed in constant pressure molecular dynamics runs using either EAM potential. Resulting structures are shown on Fig. 2b and Fig. 3b for vacancy and interstitial respectively. The final structures are highly similar to those obtained with EAM2 potential, that is no delocalization occurs and both vacancy and interstitial are compact.

The formation energies for copper vacancies and interstitials at the interface as calculated with the two potentials and DFT are listed in Table I. It should be noted that the values cannot be directly compared. The reason for this is the difference in defect energies of pure copper that will carry over to the formation energies of defects near the interface. Similarly, these values cannot be compared to the ones calculated using a larger unit cell, firstly because of possible defect-defect interaction in neighboring cells due to periodic boundary conditions and secondly on account of the probable errors introduced by approximating the large cell as outlined above. Moreover, since EAM1 does not predict formation of vacancies, but pairs of extended jogs, it can be argued that the term “vacancy formation energy” does not apply, hence making the comparison meaningless.

## Discussion

Our results demonstrate that different empirical potentials can lead to contrasting results when the structures studied are substantially different from those used for the fitting procedure. Composing an alloy potential from two single element EAM potentials involves fitting a single function relating distance between two different species of atoms to the energy. Since the data used to fit EAM1 depends only on a small discrete set of distances, this function is also well defined only at the particular points. At the same time the distribution of distances between atoms at a semicoherent interface forms a complex and nearly continuous spectrum. It follows that by using this kind of limited data set, the energy of the interface and hence the formation energies of defects can take nearly arbitrary values limited only by the choice of a functional form.

The same method of fitting as described above for EAM1 has also been used to “tune” the potentials to yield different enthalpies of mixing and to demonstrate how this would affect the behavior of vacancies and interstitials^15^,^16^. While this method is a neat example how simulations can facilitate investigating scenarios that are impossible to achieve in experiments, having a too small fitting database might lead to incorrect conclusions.

**Figure 4:**
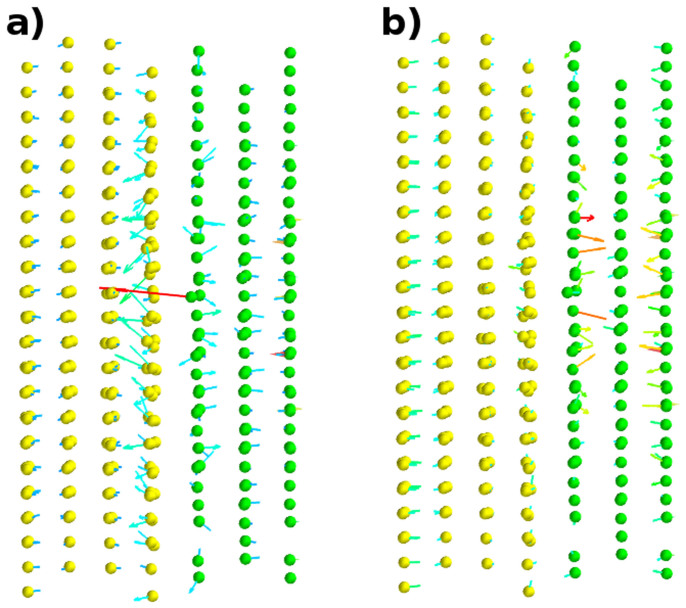
Difference between DFT and MD forces (in arb. units) on atoms for DFT-relaxed interface structure with single copper vacancy. (a) and (b) show the forces obtained using EAM1 and EAM2 respectively. EAM1 produces significantly larger forces on copper atoms near the interfacial plane compared to those of DFT and EAM2. Copper atoms - yellow, niobium atoms - green.

Another approach to obtain alloy potentials is to fit forces instead of alloy formation energies to reproduce those obtained from first-principles calculations as proposed by Ercolessi and Adams. This has been shown to lead to greater accuracy and transferability of the potentials^17^. Figure 4 shows the forces on atoms for the relaxed DFT structure with one vacancy calculated using both EAM1 and EAM2. The forces predicted by the latter differ predominantly at free surfaces and for niobium atoms. It has been shown that it is quite hard or even impossible to accurately reproduce DFT forces for niobium using EAM^18^, suggesting that some limitations of empirical potentials could not be overcome by just increasing the fitting database. With EAM1 the difference between DFT and MD forces for the first copper layer, where the vacancy is located, is much larger which leads to reconstruction of the layer and delocalization of the vacancy.

To summarize, we have shown using ab-initio techniques that single compact point defects can exist at semicoherent metal-metal interfaces without delocalization contrary to the results of previous studies. This result will likely have an impact on the behavior of defect migration, clustering and recombination, and suggest the need of further investigations. In addition, we have compared results from two EAM potentials and provided an explanation for the discrepancy between these, which can be attributed to different fitting strategies and intrinsic transferability limitations of empirical potentials.

## Methods

All DFT calculations were done using plane-wave pseudopotential code Vienna Ab initio Simulation Package (VASP)^19^,^20^,^21^,^22^ with supplied PAW pseudopotentials^23^ and GGA-PBE approximation^24^,^25^. For niobium the semi core *p* states were treated as valence. The cutoff energy for the plane waves was 273.214 eV. Single k-point (Γ-point) was used and smearing was handled by 1st order Methfessel-Paxton scheme^26^ with width of *σ* = 0.01 eV. Atomistic simulations were performed with classical molecular dynamics code LAMMPS^27^. Figures of atomic structures were created with scientific visualization and analysis software OVITO^28^.

## Author Contributions

M.K and A.A. were responsible for project planning and execution. E.M. and A.T. carried out the calculations. E.M. wrote the paper. E.M., A.T. and A.C. analyzed and interpreted the results. All authors contributed to the manuscript preparation, discussions and results.

## Figures and Tables

**Table 1:** Formation energies of copper vacancies and interstitials at the interface in eV. EAM1 tends to underestimate while EAM2 overestimates the formation energies. Values in parentheses represent the same energies for bulk copper. It should be emphasized, that a) the energies calculated do not truly represent formation energies in dilute limit due to the small number of atoms in simulation cell, and b) care must be taken when comparing DFT and MD values because of the differences in formation energies in pure copper

Structure	EAM1^a^	EAM2^b^	DFT^c^
Vacancy	0.18 (1.26)	0.72 (1.27)	0.38 (1.17)
Interstitial	1.07 (3.27)	1.52 (3.09)	1.13 (3.86)

^a^Potential by Demkowicz et al^11^.

^b^Potential by Zhang et al^12^.

^c^This work.
